# Corrosion Behavior of an Mg_2_Sn Alloy

**DOI:** 10.3390/ma15062025

**Published:** 2022-03-09

**Authors:** Zuzana Gabalcová, Peter Gogola, Žaneta Gerhátová, Marián Palcut

**Affiliations:** Faculty of Materials Science and Technology in Trnava, Institute of Materials Science, Slovak University of Technology in Bratislava, J. Bottu 25, 917 24 Trnava, Slovakia; zuzana.gabalcova@stuba.sk (Z.G.); peter.gogola@stuba.sk (P.G.); zaneta.gerhatova@stuba.sk (Ž.G.)

**Keywords:** corrosion, oxidation, magnesium, tin, Mg_2_Sn

## Abstract

In the present work, the corrosion behavior of the Mg_2_Sn alloy (Mg_66.7_Sn_33.3_, concentration in at.%) has been studied. The alloy was prepared from high purity Sn and Mg lumps by induction melting in argon. The alloy was composed of intermetallic Mg_2_Sn with a small amount of Mg_2_Sn + (Sn) eutectic. The corrosion behavior was studied by hydrogen evolution, immersion, and potentiodynamic experiments. Three aqueous solutions of NaCl (3.5 wt.%), NaOH (0.1 wt.%) and HCl (0.1 wt.%) were chosen as corrosion media. The alloy was found to be cathodic with respect to metallic Mg and anodic with respect to Sn. The corrosion potentials of the Mg_2_Sn alloy were −1380, −1498 and −1361 mV vs. sat. Ag/AgCl in HCl, NaCl and NaOH solutions, respectively. The highest corrosion rate of the alloy, 92 mmpy, was found in aqueous HCl. The high corrosion rate was accompanied by massive hydrogen evolution on the alloy’s surface. The corrosion rate was found to decrease sharply with increasing pH of the electrolyte. In the NaOH electrolyte, a passivation of the alloy was observed. The corrosion of the alloy involved a simultaneous oxidation of Mg and Sn. The main corrosion products on the alloy surface were MgSn(OH)_6_ and Mg(OH)_2_. The corrosion mechanism is discussed and implications for practical applications of the alloy are provided.

## 1. Introduction

Magnesium–tin alloys are promising candidates for high temperature applications [1]. Tin has low diffusivity and reasonable solid solubility in Mg [1,2]. The diffusivity of Sn in Mg is approximately an order of magnitude lower than that of Zn [3,4]. Due to the large differences in the solid solubility of Sn in Mg at high and low temperatures, intermetallic Mg_2_Sn can easily precipitate during solidification [2,3]. The Mg_2_Sn precipitates have a low grain boundary sliding rate in high temperature environments [5]. In addition to anti-creep properties, the alloying of Mg with Sn also improves the tensile strength [6], castability [7], and extrusion properties of Mg alloys [8]. Moreover, the thermal conductivity of alloys decreases with increasing Sn content [9]. Therefore, Mg–Sn alloys have been considered as phase change materials for thermal energy storage [10].

Sn is a relatively non-toxic element. As such, Mg–Sn alloys can be used as bio-absorbable implants [11,12,13,14]. The corrosion behavior of Mg–Sn alloys is related to the amount of Mg_2_Sn phase precipitated during solidification [15]. The presence of Mg_2_Sn phase in the Mg alloy promotes galvanic corrosion and accelerates the alloy corrosion rate [15,16]. If the tin concentration in Mg–Sn alloys is low, the Mg_2_Sn phase is not precipitated. If most of the tin is dissolved in the Mg matrix, the alloy self-corrosion rate can be significantly reduced [17]. Mg–Sn alloys can produce Sn-rich passive layers during immersion in chloride-containing aqueous electrolytes [18]. The passive layer provides sluggish hydrogen evolution kinetics in a manner comparable to Mg–As and Mg–Ge alloys [19].

The Mg_2_Sn phase is commonly observed in various alloy systems. In our previous study of Zn–Mg–Al–Sn alloy castings, the Mg_2_Sn phase was precipitated in addition to (Zn) and (Al) solid solutions, as well as Mg_2_Zn_11_ and MgZn_2_ intermetallic phases [20]. The phase was found to be nobler than Mg_2_Zn_11_ and MgZn_2_. Furthermore, we observed a local de-alloying of Mg_2_Sn particles [21]. The Mg concentration in Mg_2_Sn was gradually reduced during corrosion, resulting in the production of Sn-rich particles in place of Mg_2_Sn particles. The presence of the Mg_2_Sn phase in Mg–Sn alloys is often critical for the corrosion resistance of the alloys [15,16,17]. It is known to promote galvanic corrosion of the Mg matrix and accelerate the alloy corrosion rate. The corrosion mode and the corrosion rate are associated with the volume of the Mg_2_Sn phase and the concentration of tin in the matrix [22]. The Sn addition increases the overall dissolution rate of the alloys but also promotes passivity. The overall corrosion behavior primarily depends on the amount of Mg_2_Sn intermetallic [23]. It notably increases the H_2_ evolution rate and functions as pitting corrosion initiation sites in Cl-containing electrolytes. As the area fraction of grain boundaries increases, the H_2_ evolution rate is also accelerated. In contrast, Sn dissolved in the Mg matrix decreases the H_2_ evolution rate [16]. As Sn has a low exchange current density, it does not enhance the cathodic kinetics.

The maximum solubility of Sn in Mg is ~3 at.% [1,2]. The wide range of compositional solid solubility constitutes an opportunity for designing Mg–Sn alloys with tunable electrochemical properties. Most previous studies have investigated the effect of Sn concentration in selected solid solution Mg_100x_–Sn_x_ alloys (x = 1–3 at.%) on their electrochemical performance in chloride-containing electrolytes [15,16,17,18,19,20,21,22,23,24]. Nevertheless, alloys with Sn concentration higher than 3 at.% have been significantly less explored. 

Sn forms eutectic with Mg_2_Sn at 91 at.% Sn. The eutectic has a relatively low melting point (472 K, [2]). As such, Sn-rich Mg–Sn alloys are potential candidates for lead-free soldering. The corrosion resistance of an Mg_9.5_Sn_90.5_ alloy has been reported to be lower compared to conventional Mg alloys [25]. It has been observed that the presence and distribution of the Mg_2_Sn phase in the Sn rich matrix promotes the formation of Sn/Mg_2_Sn galvanic pairs. The galvanic couples directly influence the hydrogen evolution rate. The microstructure formed by more refined fibers presents a surface with the circularly shaped Mg_2_Sn phase evenly distributed in the Sn matrix. This microstructural configuration leads to the formation of tin oxide corrosion products that inhibit the evolution of H_2_ [25]. On the other hand, the reaction kinetics for the coarse microstructure alloy is increased, resulting in a higher hydrogen evolution rate. 

Mg_2_Sn is the only intermetallic phase present in the Mg–Sn phase diagram [2]. The compound has an inverted fluoride crystal structure with Mg occupying the F positions and Sn occupying Ca positions [26]. A large amount of small-sized Li can be reversibly inserted into the crystal lattice of mechanically alloyed Mg_2_Sn [27]. Therefore, the material has been studied as lithium storage intermetallic compound. Unlike isostructural Mg_2_Si, an alloying reaction between Mg and Li does not occur [28]. Furthermore, a recent study showed that pure Sn does not produce an enhanced cathodic kinetics [29]. These properties make Mg–Sn alloys ideal candidates for battery anodes [30,31,32,33]. The bulk Mg_2_Sn phase can be de-alloyed, thereby producing a nanostructured Sn for high performance battery anodes [34]. Mg_2_Sn is also a promising thermoelectric material that can directly convert waste heat into electricity [35]. Its thermoelectric properties can be optimized through point defect engineering [36]. 

Despite its numerous practical applications, the corrosion behavior of Mg_2_Sn has not been investigated yet. In the present work, our objective is to investigate the corrosion behavior of an Mg_2_Sn alloy (Mg_66.7_Sn_33.3_, concentration in at.%). The alloy was prepared by induction melting of pure elements in argon. The corrosion resistance has been studied in three different aqueous solutions: 3.5 wt.% NaCl, 0.1 wt.% HCl and 0.1 wt.% NaOH. The solutions were carefully chosen to investigate the effects of H^+^ and Cl^-^ concentrations on the corrosion rate. Our aim is to provide fundamental corrosion data for the Mg_2_Sn intermetallic phase in various environments. The results might be useful for scientists working with Mg alloys in different applications including biomedicine, electrochemistry and electronics. 

## 2. Materials and Methods

The Mg_2_Sn alloy was prepared by melting Sn and Mg lumps (purity of 99.99 wt.%) in argon. The metals were weighed in respective concentrations and placed in an alumina crucible (Brisk, Tábor, Czech Republic). The crucible was positioned inside a vacuum induction furnace (Rajmont, Hradec Králové, Czech Republic). Before melting, the furnace chamber was evacuated by rotary pump and flushed with high purity argon (99.999 vol.%). The flushing was repeated three times. The Mg and Sn granules were induction heated to 800 °C (1073 K, controlled by pyrometer) and held at this temperature for 30 s. The alloy was subsequently cooled by turning the furnace off. The alloy was solidified to form cast cylinders. The cast cylinders were cut with a diamond saw and mounted in epoxy resin. The samples were metallographically prepared by grinding and polishing. The final polishing step was carried out using a 1 μm monocrystalline diamond. 

To study the phase constitution, the alloy was powdered and crushed in a mortar. The powder was used to reduce the influence of the casting texture on the XRD pattern. The alloy powder was investigated using the PANalytical Empyrean X-ray diffractometer (XRD, Malvern Panalytical Ltd., Malvern, United Kingdom). A Ni-filtered CuK_α1_ radiation (*λ* = 1.54060 nm) was used as the excitation source. The diffraction parameters are listed in Table 1. The scattering angle was measured using Bragg–Brentano geometry. The XRD patterns were further analyzed using the PANalytical Xpert High Score program (HighScore Plus 3.0.5 version) with the ICSD FIZ Karlsruhe database. 

A JEOL JSM7600 F scanning electron microscope (SEM) (JEOL Ltd., Tokyo, Japan) was used to examine the microstructure of the alloy. A voltage of 15 kV was applied to accelerate the electron beam during the measurements. The chemical composition of the Mg_2_Sn alloy was studied with an energy dispersive X-ray spectrometer (EDX) integrated within SEM. The operation of the EDX detector was controlled by INCA software (Oxford Instruments NanoAnalysis, Bucks, UK).

The corrosion experiments were carried out in three aqueous solutions of NaCl (3.5 wt.%), HCl (0.1 wt.%) and NaOH (0.1 wt.%). By choosing the electrolytes, we have been able to investigate the effects of both pH and Cl^-^ concentration on the corrosion rate. NaCl (3.5 wt.%) is a standard saline solution used for corrosion testing of metals [37]. HCl is a strong reducing acid. It was chosen due to its low pH, high Cl concentration and biomedical importance. Dilute HCl is found in the gastric tract, where it helps to ease digestion and protect against infection [38]. NaOH is a strong base. The dilute NaOH solution was chosen due to its high pH. Magnesium alloys are known to produce passive layers in alkaline environments. The solutions were prepared immediately before the experiment by dissolving the weighted amounts of the electrolyte in deionized water (conductivity < 20 μS). The pH of the electrolyte was measured with a handheld pH meter (Thermo Fisher Scientific, Waltham, MA, USA) at room temperature. 

The corrosion experiments started with the hydrogen evolution method. The volume of hydrogen evolution of the alloy was measured at room temperature. The alloy was placed in a glass beaker and covered with the electrolyte. The exposed surface area was 1.45 cm^2^. A funnel was positioned over the sample to collect the generated hydrogen. The end of the funnel was placed in an inverted burette pre-filled with electrolyte. The volume of the electrolyte in the burette was leveled to zero at the beginning of the experiment. The volume change of the solution corresponded to the hydrogen volume that evolved from the alloy during immersion. The experimental error in volume reading given by the burette gauge was ±0.1 mL. The hydrogen volume was recorded as a function of time. After the experiment, the hydrogen evolution rate and corrosion rate of the alloy were calculated.

Electrochemical experiments were conducted in a 500 mL glass beaker filled with the aqueous electrolyte. A standard three-electrode arrangement was used for the measurements. The working electrode was the polished surface of the Mg_2_Sn alloy with an exposed area of 5.3 cm^2^. A saturated Ag/AgCl electrode served as a reference electrode. The counter electrode was a platinum foil (4 cm^2^). Experiments were carried out at room temperature. The solutions were prepared immediately before the experiment by dissolving the respective amount of the electrolyte in deionized water (conductivity < 20 µS). The electrolytes were not de-aerated before the experiment to simulate real environmental conditions. The progress of the reaction progress was monitored by a PGU 10 V-1A-IMP-S potentiostat/galvanostat (Jaissle Electronic Ltd., Waiblingen, Germany). 

Initially, an open circuit potential of the alloy (OCP) was recorded for over 30 min. The experimental error in the determination of the OCP was ±1 mV. After the OCP measurement, a potentiodynamic polarization of the alloy started. The polarization began at potentials of −500 mV vs. OCP and continued in a positive direction. The polarization experiment was stopped when currents greater than 10^3^ A m^−2^ were achieved. The resulting polarization curves were plotted in semilogarithmic coordinates. The curves were analyzed by Tafel extrapolation [39]. 

To study the phase constitution of the corrosion products, immersion experiments were conducted. The alloy castings were first powdered and then immersed in the respective solution for several days. The corrosion products were crushed in a mortar and the resulting fine powders were used for the XRD measurements. Quantitative results were obtained from XRD patterns using Rietveld refinement integrated in the software MAUD version 2.84 [40]. The program uses an asymmetric pseudo-Voight function to describe the experimental peaks. The instrument broadening was determined by measuring the position of the NIST660c LaB_6_ line and the line broadening standard (The National Institute of Standards and Technology, Gaithersburg, MD, USA). It was introduced to the Rietveld refinement program via the Caglioti equation.

## 3. Results and Discussion

### 3.1. Microstructure and Phase Constitution of the Alloy

**Figure 1:**
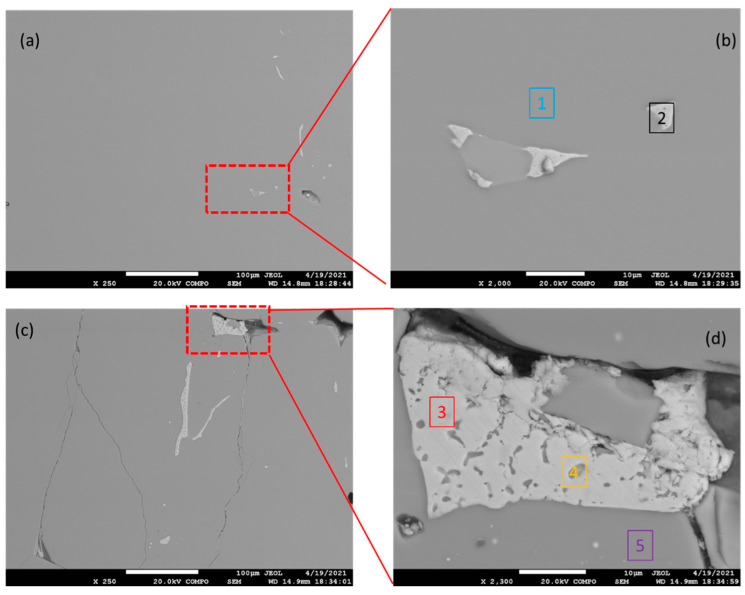
BSEM images of the as-cast microstructure of the Mg_2_Sn alloy. The edge of the sample is shown in (**a**,**b**); center of the sample is shown in (**c**,**d**). The chemical composition of points 1–5 is given in Table 2.

The microstructure of the as-cast Mg_2_Sn alloy is given in Figure 1. The microstructure was checked at several sites across the 30 mm diameter of the sample. Figure 1a,c show the alloy constitution at the edge and near the center, respectively. The images were acquired in a backscatter electron (BSEM) mode. As such, they provide an element resolution. The EDX analyses of points 1–5 is shown in Table 2. The alloy was formed mainly by Mg_2_Sn, as indicated by the EDX analysis. A limited number of Sn-rich particles were also found. The Sn-rich particles appear bright in Figure 1a,c. The Sn-rich particles are most probably a part of Mg_2_Sn-(Sn) eutectic, as indicated in Figure 1d. The Mg_2_Sn microstructure component is darker as it contains less Sn compared to the eutectic. Several occasional cracks and pull-outs were also observed in the alloy microstructure. The defects were preferentially located in the Mg_2_Sn microstructure component. The cracks and pull-outs probably originated during cooling and/or metallographic preparation because of the brittle nature of the Mg_2_Sn intermetallic phase. 

**Figure 2:**
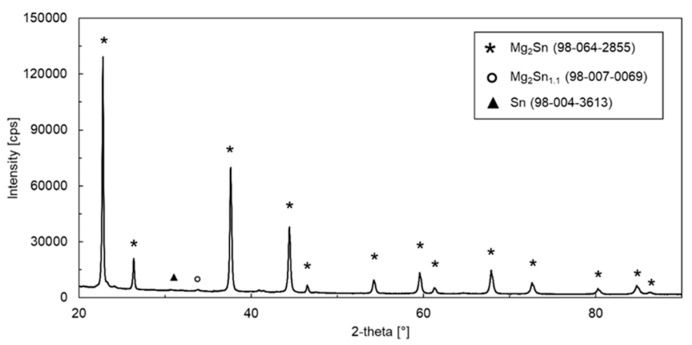
XRD pattern of as-cast Mg_2_Sn alloy.

The phase constitution of the powdered alloy was studied by XRD. The XRD pattern of the alloy is shown in Figure 2. The most intensive peaks were found to correspond to the Mg_2_Sn intermetallic. The minor peaks corresponded to Mg_2_Sn_1.1_ and Sn. All identified phases are summarized in Table 3. An approximate volume fraction of each phase, calculated by quantitative Rietveld analysis, is also included in Table 3. The prepared alloy was nearly single phase. The volume fraction of Mg_2_Sn was 95% (Table 3). In addition to Mg_2_Sn, a small amount of Mg_2_Sn_1.1_ has been found. This phase contains a certain amount of Mg vacancies, making the Mg:Sn molar ratio lower than 2 [41]. Mg_2_Sn_1.1_ could have been formed due to Mg evaporation. The volume fraction of (Sn) was only 0.25%.

$\mathrm{Fm}\bar{3}$$P\bar{3}$As Mg_2_Sn was the major microstructural component, an isotropic size-strain model was applied only to this intermetallic. Minor discrepancies between the nominal and measured peak intensities were found. These discrepancies were corrected using the spherical harmonic functions with fiber symmetry. A weighted profile *R*-factor (*R*_wp_) of 5.6% was achieved.

### 3.2. Hydrogen Evolution Experiments

**Figure 3:**
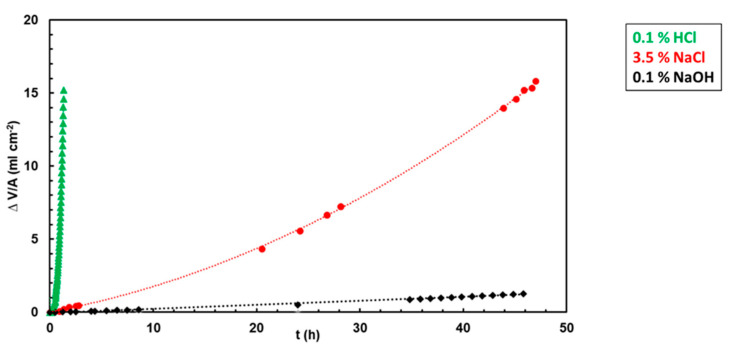
Hydrogen evolution volumes of the Mg_2_Sn alloy in 0.1% HCl, 3.5% NaCl and 0.1% NaOH.

A hydrogen evolution rate of the as-cast Mg_2_Sn alloy was measured in different electrolytes. The experiments were carried out in aqueous solutions of NaCl (3.5 wt.%), HCl (0.1 wt.%) and NaOH (0.1 wt.%). The polished alloy surface was placed in a glass beaker and covered with the electrolyte. A funnel was placed over the sample to collect the generated hydrogen. The end of the funnel was positioned in an inverted burette pre-filled with electrolyte. The hydrogen evolution volumes, recorded for up to 48 h, are compared in Figure 3. In HCl, a massive hydrogen generation was observed. The massive hydrogen generation is caused by the following reaction:

Mg + 2H^+^ → H_2_ + Mg^2+^ (1)

In this reaction, H^+^ cations are reduced to gaseous H_2_ at the expense of metallic magnesium. The excessive hydrogen generation has also been observed during OCP measurements and will be discussed in the next chapter. 

A hydrogen evolution rate, *V_R_*(*H*_2_), was calculated from the maximum hydrogen volume by the following formula:

(2) $$V_{R}\left(H_{2}\right)=\frac{\Delta V_{H}}{A.t}\;$$

**Figure 4:**
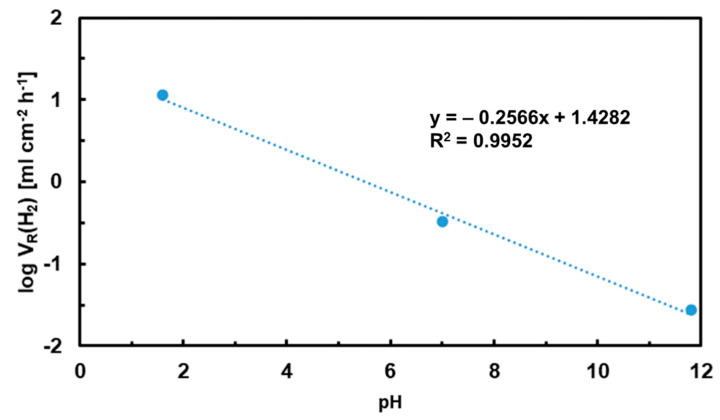
Hydrogen evolution rates of the as-cast Mg_2_Sn alloy as a function of the pH of the electrolyte.

In Equation (2), ∆*V_H_* is the hydrogen volume, *A* is the exposed surface area and *t* is the total immersion time. Hydrogen evolution rates in different electrolytes are compared in Figure 4. Please note the logarithmic scale on the *y*-axis. Figure 4 shows that the hydrogen evolution rate in HCl is more than one order of magnitude higher compared to NaCl and more than two orders of magnitude higher compared to NaOH. In chloride-containing HCl, MgCl_2_ forms as a reaction product. MgCl_2_ is water-soluble. As such, it is not capable of producing a passive layer on the alloy surface. Therefore, the alloy corrosion progresses in HCl at a high rate. 

The hydrogen evolution rate slows down in NaCl and NaOH solutions (Figure 3). In NaCl and NaOH, the concentration of H^+^ is low. Therefore, hydrogen is more likely to be produced by the following reaction [24,42]:

Mg + 2H_2_O → H_2_ + Mg(OH)_2_ (3)

As a result of Reaction (3), magnesium hydroxide is produced. Magnesium hydroxide may also be produced by the following reaction:

2Mg + O_2_ + 2H_2_O → 2Mg(OH)_2_ (4)

In Reaction (4), no gaseous hydrogen is generated. Magnesium hydroxide is a water insoluble solid [42]. It forms a passive layer on the surface. Therefore, a lower hydrogen generation rate was observed in NaCl and NaOH, respectively. The passive layer may significantly retard alloy corrosion and lower the amount of hydrogen produced. 

The recorded hydrogen generation rate in NaCl was higher compared to NaOH (Figure 3). In chloride-containing electrolytes, the Cl^−^ anions may react with Mg(OH)_2_ according to the following reaction:

Mg(OH)_2_ + 2Cl^−^ → MgCl_2_ + 2 OH^−^ (5)

During Reaction (5) a water-soluble MgCl_2_ is produced. As a result of Reaction (5), the passive layer is weakened, which leaves a naked Mg alloy surface susceptible to further attack. Therefore, the hydrogen generation rate in Cl-containing NaCl was higher compared to that in NaOH.

### 3.3. Electrochemical Corrosion Experiments (OCP, Polarization)

**Figure 5:**
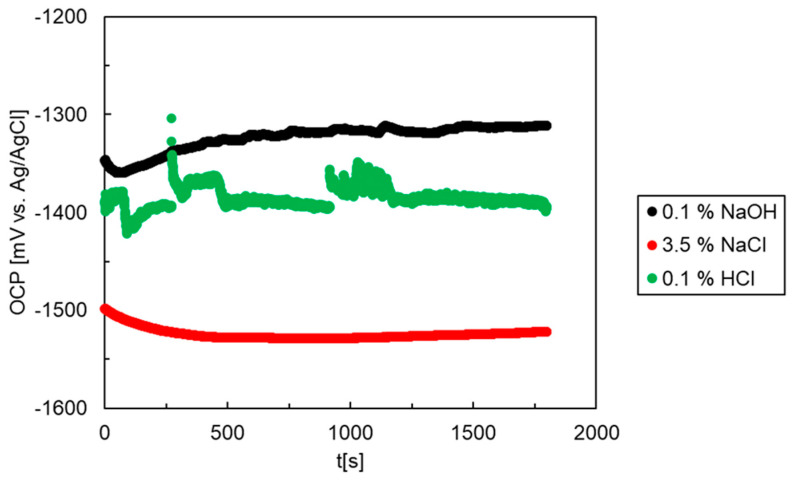
Open circuit potentials of the Mg_2_Sn alloy in aqueous NaOH (0.1 wt.%), NaCl (3.5 wt.%) and HCl (0.1 wt.%) solutions.

The corrosion behavior of the as-cast Mg_2_Sn alloy was further investigated by electrochemical methods. The solutions were prepared by weighing the respective amounts of NaCl, NaOH and HCl and dissolving the electrolytes in de-ionized water. Immediately after the alloy’s immersion in the electrolyte, an OCP was measured. The OCPs of the alloy recorded over 30 min in the three different solutions are presented in Figure 5. In HCl, several irregular oscillations and transient bursts have been observed. The bursts were caused by massive hydrogen evolution. The hydrogen bubbles entered the Haber-Luggin capillary and caused several transient disruptions of the electrical circuit. Therefore, the transient bursts of the OCP in this electrolyte could not be experimentally avoided.

The OCPs of the Mg_2_Sn alloy increase in the following order:

NaCl ˂ HCl ˂ NaOH(6)

The lowest OCP is found in NaCl aqueous solution. In this electrolyte, an initial decrease in the OCP has been observed (Figure 5). The initial decrease was followed by stabilization at times greater than 10 min (600 s). The OCP of the Mg_2_Sn alloy in HCl was higher compared to NaCl. Aqueous solutions of NaCl, HCl and NaOH have different concentrations of H^+^ and Cl^-^ ions. The observations presented above indicate that the OCPs of the Mg_2_Sn alloy are influenced by the concentration of chloride anions. The highest concentration of chlorides was present in NaCl, followed by HCl. 

In NaOH, the highest OCPs have been found, indicating a higher nobility of the Mg_2_Sn alloy in this electrolyte. In NaOH, the OCP was observed first to decrease and later slightly increase with time (Figure 5). This observation indicates a possible passivation of the Mg_2_Sn alloy in this electrolyte. 

**Figure 6:**
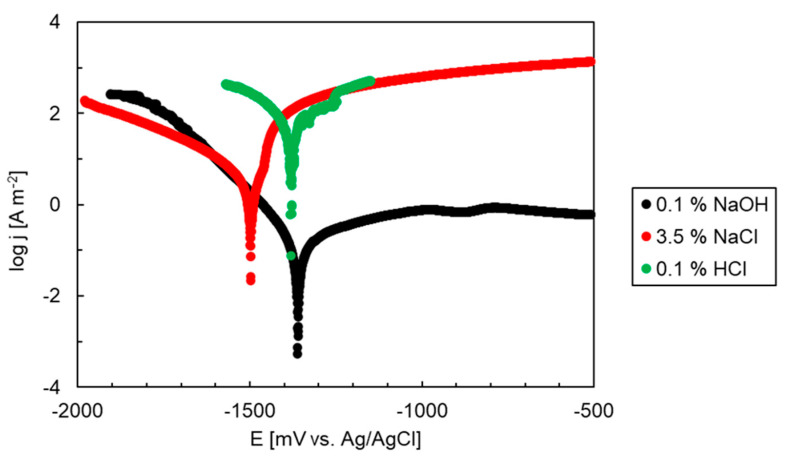
Polarization curves of the as-cast Mg_2_Sn alloy in different electrolytes.

The Mg alloys are prone to pitting corrosion in Cl-containing electrolytes [42,43]. The corrosion behavior of the Mg_2_Sn alloy was therefore further investigated by potentiodynamic polarization. After the OCP measurement, a polarization scanning from −2000 mV to −500 mV (Ag/AgCl) was performed using a sweeping rate of 1 mV s^−1^. The resulting polarization curves of the Mg_2_Sn alloy are presented in Figure 6. The curves have a single corrosion minimum observed at ~−1500 mV (vs. Ag/AgCl). The corrosion minimum corresponds to anodic oxidation of magnesium. After passing the minimum, a steady increase of current density was observed. The current density increase was further followed by either stabilization (NaOH) or an abrupt increase at larger electrode potentials (HCl, NaCl).

The experimental polarization curves were analyzed by Tafel extrapolation [39,44]. The extrapolated corrosion potentials and the corrosion current densities are collected in Table 4. The corrosion potentials of the Mg_2_Sn alloy in NaCl, HCl and NaOH solutions correspond to open circuit potentials measured in the previous experiment. The corrosion currents were found to increase in the following order:

NaOH ˂˂ NaCl ˂˂ HCl(7)

The shift of *j*_corr_ to higher values was remarkable and corresponded to an approximately one order of magnitude difference between the electrolytes. The highest corrosion rate was found in HCl aqueous solution. This result is interesting since HCl (0.1 wt.%) has a lower concentration of chlorides compared to NaCl (3.5 wt.%). Therefore, it is the pH of the solution that influences the overall corrosion rate.

**Figure 7:**
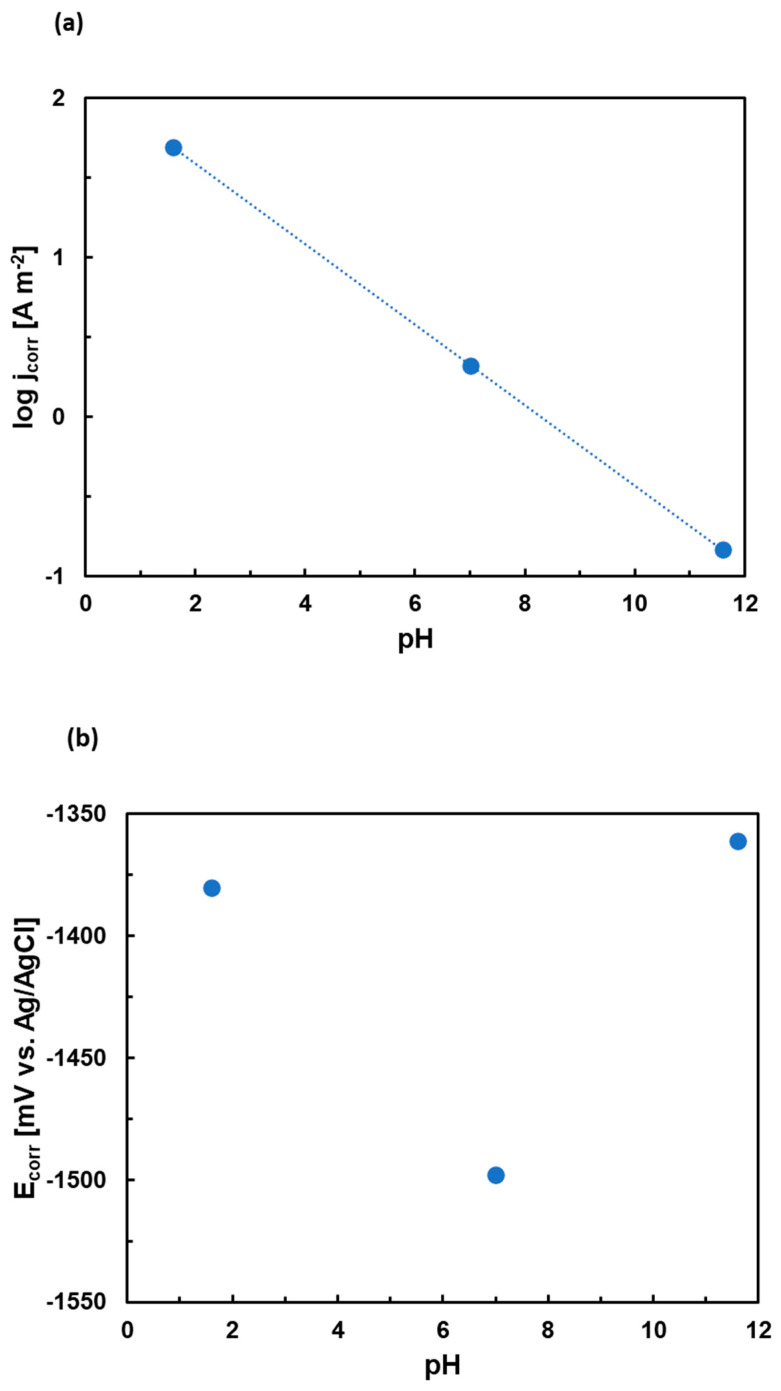
The dependence of corrosion currents (**a**) and corrosion potentials (**b**) of the as-cast Mg_2_Sn alloy on the pH of the solution.

The dependence of corrosion parameters on pH is presented in Figure 7. The corrosion current drops sharply with increasing pH of the electrolyte. The dependency of corrosion potentials on pH is less straightforward. It follows the same trend as OCP, i.e., the highest corrosion potentials are found in NaOH. It is possible that a passive layer forms in this electrolyte. The passive layer of Mg alloys is preferably formed in alkaline electrolytes according to the E-pH diagram of magnesium [19,45]. The layer may have contributed to the observed ennoblement of the Mg_2_Sn alloy in NaOH solution.

**Figure 8:**
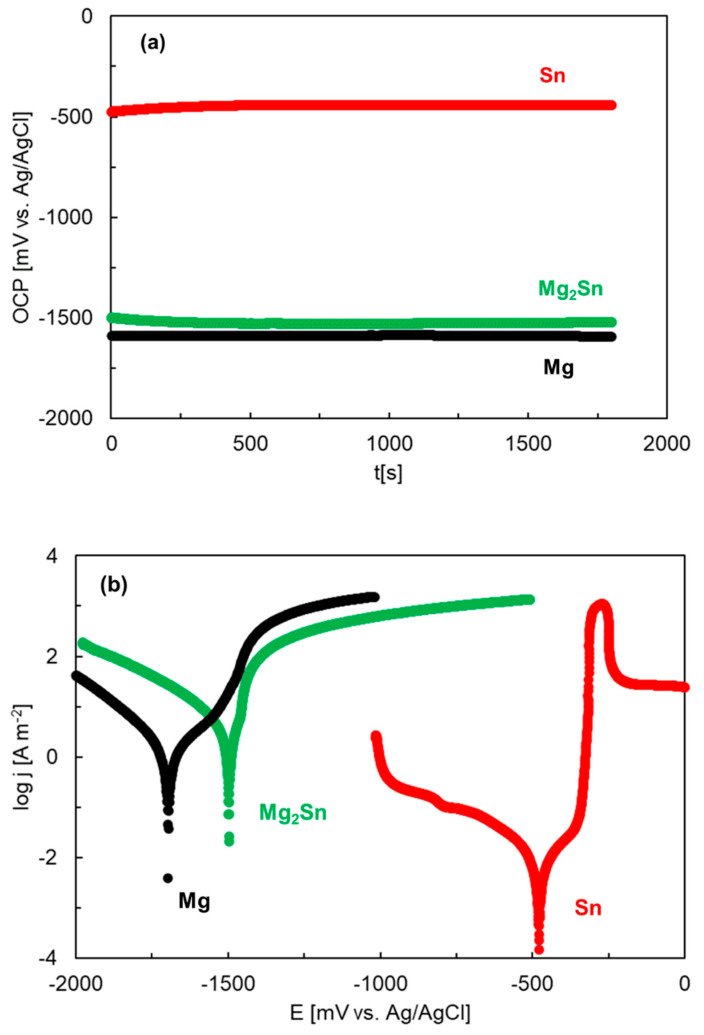
Open circuit potentials (**a**) and polarization curves (**b**) of Mg, Sn and Mg_2_Sn. The experiments were conducted in aqueous NaCl solution (3.5 wt.%) at room temperature.

For binary Mg–Sn alloys, alloying with Sn has been reported to cause an anodic activation [19,42]. Therefore, we have compared the corrosion behavior of the Mg_2_Sn alloy with that of pure Mg and pure Sn. The results, measured in aqueous NaCl solution, are compared in Figure 8. The open circuit potentials increase in the following order:

Mg ˂ Mg_2_Sn ˂˂ Sn (8)

The corrosion potential of Mg_2_Sn is slightly higher compared to Mg. As such, Mg_2_Sn is cathodic with respect to Mg. Sn has a remarkably high OCP compared to both Mg and Mg_2_Sn. Mg_2_Sn is anodic with respect to Sn. In the present case, the Mg_2_Sn alloy was composed of Mg_2_Sn accompanied by a small amount of Mg_2_Sn + Sn eutectic. We also observed that Mg_2_Sn had a higher corrosion current density compared to Mg (Figure 8b). The tin-rich eutectic may have anodically activated the surrounding Mg_2_Sn matrix. The presence of Sn in the eutectic probably promoted the formation of Sn/Mg_2_Sn galvanic couples that influenced the anodic oxidation of Mg_2_Sn.

Mg_2_Sn has a cubic crystal structure [26]. It adapts a fluorite (CaF_2_) structure with Mg occupying the fluorine positions and Sn occupying the calcium positions. In this structure, the smaller Mg atom is tetrahedrally coordinated by four Sn atoms. The larger Sn is octahedrally coordinated by eight Mg atoms. The crystal cohesion is probably lowest in (111) planes [46]. The (111) plane thus probably constitutes the cleavage plane. The (111) plane is formed by Sn atoms, with Mg situated off this plane. The formation of a corrosion product between the cleavage planes may result in a significant tension that may mechanically break the crystal. The Mg atoms are prone to corrosion attack. It has been reported that Mg_2_Sn may be de-magnesiated, thus producing a Sn-rich porous structure [34]. Using finite element modeling, it has been found that large stresses develop during continuous electrochemical de-alloying. The stresses induce pulverization of the de-alloyed Mg_2_Sn and lead to a formation of nanostructured Sn. An interface between the de-magnesiated shell and the magnesiated core forms within the Mg_2_Sn grain. Progressive de-magnesiation results in a movement of the interface between the growing de-magnesiated shell of Sn and shrinking magnesiated core of Mg_2_Sn. In the end, the generated chemo-mechanical stresses induce cracks in the material and lead to pulverization [46,47].

**Figure 9:**
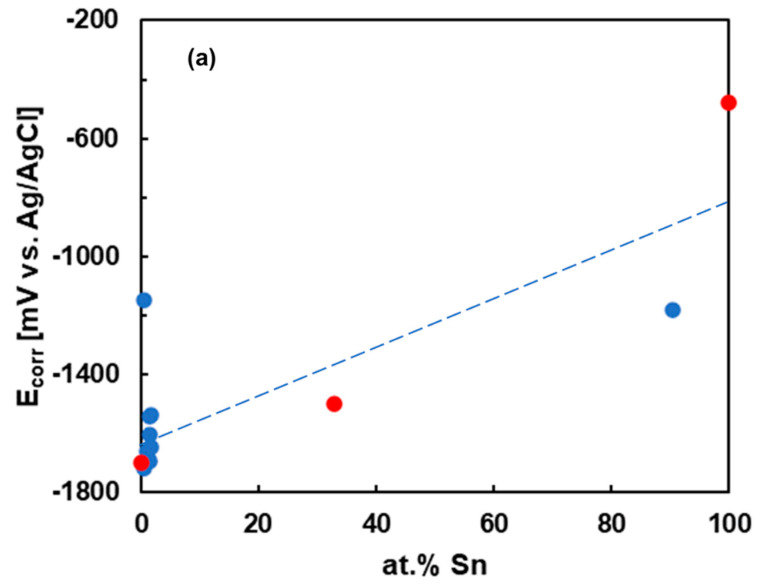

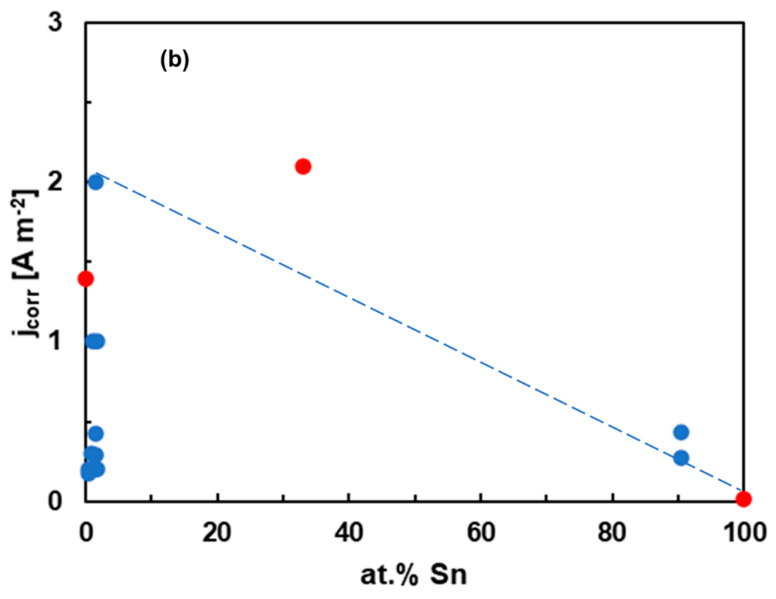
Corrosion potentials (**a**) and corrosion current densities (**b**) of Mg–Sn alloys in 3.5% NaCl. The present data are given in red. The dashed lines are guides to the eyes only.

To further probe the effects of chemical composition, we have compared the corrosion currents and corrosion potentials of the Mg_2_Sn alloy with those of previously studied Mg–Sn alloys [15,16,17,24,25,48,49]. The dependence of the electrochemical parameters (*E*_corr_, *j*_corr_) on the Sn atomic fraction is compared in Figure 9a,b. As most data in the literature were reported for 3.5 wt.% NaCl, only this electrolyte is presented in Figure 9. The corrosion potentials increase with increasing tin concentration. This observation indicates that Sn contributes to an ennoblement of the Mg–Sn alloys at larger Sn concentrations. The corrosion current of the present alloy is higher compared to those of previously studied Mg–Sn alloys with significantly lower Sn atomic fractions [15,16,17,50,51]. It can be observed that Sn addition contributes to anodic activation, previously reported for Mg–Sn alloys with 1–3 at.% Sn [19,42].

The comparison of data in Figure 9 has certain limitations. It must be stated that alloys with larger Sn concentrations have been significantly less explored. Therefore, they are underrepresented in Figure 9. Furthermore, the alloys compared had different microstructures and different Mg_2_Sn volume fractions. The Mg_2_Sn fraction, grain size and interphase spacing were found to influence the corrosion behavior [16,25]. A better corrosion resistance was found for more refined microstructures. To draw general conclusions, the alloys with similar microstructure and Mg_2_Sn distribution would need to be compared. Such comparison for all Mg–Sn alloy compositions is very difficult. The dashed lines in Figure 9 should be taken as guides for the eye only. 

### 3.4. Calculation of Corrosion Rates

The electrochemical parameters given in Table 4 have been used to estimate the corrosion rate of the Mg_2_Sn alloy. The corrosion rate, *CR_j_*, can be calculated from the corrosion current density, *j_corr_*, using the following formula [52]:

(9) $$CR_{j}=K\frac{j_{corr}}{\rho }E_{w}$$

In this equation, *K* is a time constant (3.27 × 10^−3^), *ρ* is the alloy density (3.6 g cm^−3^), *j_c__orr_* is the corrosion density (in μA cm^−2^), *CR_j_* is the corrosion rate (in millimeters per year, mmpy) and *E_w_* is an equivalent weight. The equivalent weight has been calculated using the following formula [52]:

(10) $$\;E_{w}=\frac{1}{\frac{z_{Mg}f_{Mg}}{A_{Mg}}+\frac{z_{Sn}f_{Sn}}{A_{Sn}}}$$

In Equation (10), *z_Mg_* and *z_Sn_* are valence states of Mg and Sn (+2 and +4, respectively), *f_Mg_* and *f_Sn_* are mass fractions of the elements in the alloy, and *A_Mg_* and *A_Sn_* are atomic masses of Mg and Sn, respectively. Furthermore, the corrosion rate, *CR_H_* can also be calculated from the hydrogen evolution rate by the following formula [51,53]

(11) $$CR_{H}=qV_{R}\left(H_{2}\right)$$

**Figure 10:**
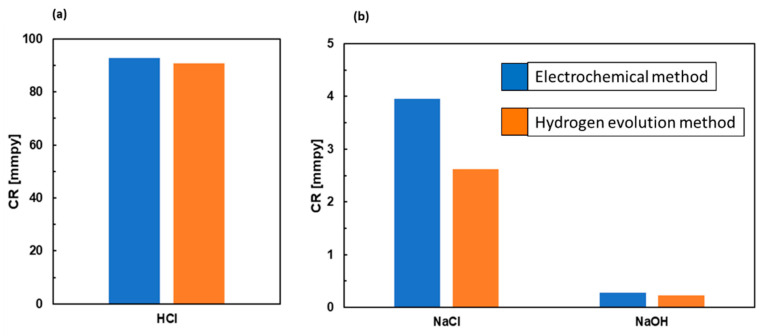
Corrosion rates of the Mg_2_Sn alloy in aqueous HCl (**a**), NaCl and NaOH solutions (**b**).

In Equation (11), *V_R_*(*H*_2_) is the hydrogen evolution rate (in mL cm^−2^ d^−1^) and *q* is a material constant. In the present case, *q* = 0.33. The corrosion rates calculated from corrosion current densities and hydrogen evolution rates are shown in Figure 10. In general, a strong agreement between the methods is observed. Furthermore, the corrosion rates obtained in aqueous NaCl are comparable to those of Mg–Sn alloys studied previously [50].

Despite a good agreement between the methods, the *CR* calculated from the corrosion current in NaCl was slightly higher compared to the rate calculated from the hydrogen evolution volume. It has been reported that the corrosion current density of magnesium and its alloys could be affected by the so-called negative difference effect (NDE, [37,38,39]). The NDE is defined as the difference between the hydrogen evolution rate under open circuit conditions and the rate associated with the hydrogen evolution reaction during anodic polarization. The NDE causes a dissolution current of magnesium alloys to increase faster than expected [54,55,56]. Therefore, the corrosion rate calculated from the corrosion current in NaCl electrolyte might have been slightly overestimated. As a result of this, the volumetric corrosion rate should be taken as more reliable.

The volumetric corrosion rate of the Mg_2_Sn alloy in NaCl is 2.5 mmpy (Figure 10). This value is comparable to the Mg–Sn alloys with low Sn atomic fractions studied previously [50]. The relatively good corrosion resistance of the Mg_2_Sn alloy, found in aqueous NaOH solution (Figure 10), is probably related to the formation of the passive layer [57]. The corrosion films formed on Mg–Sn alloys may display superior barrier properties. It has been reported that the Mg_98_Sn_2_ alloy (conc. in at.%) forms a compact passive layer over the entire alloy surface. The passive layer contributes to its superior corrosion resistance [18]. To further investigate the properties of the passive film, the microstructure and phase constitution of the corrosion products have been investigated. The results are discussed in the following chapter.

### 3.5. Microstructure and Phase Constitution of Corrosion Products

**Figure 11:**
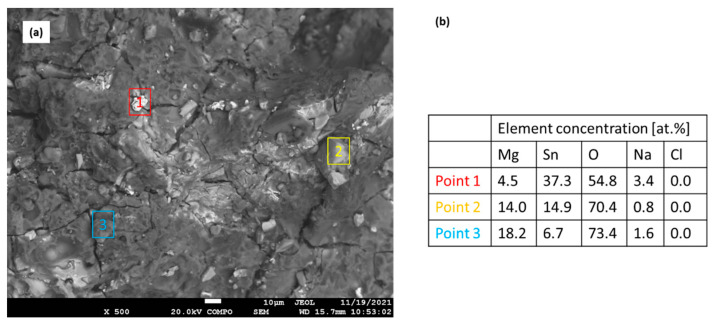
Microstructure (**a**) and chemical composition (**b**) of the corrosion product on the Mg_2_Sn alloy after potentiodynamic polarization in 3.5% NaCl.

The microstructure and chemical composition of the corrosion products formed on the Mg_2_Sn alloy after the potentiodynamic polarization in 3.5 wt.% NaCl are shown in Figure 11a. The alloy surface was covered by a thick layer of corrosion products. Figure 11a was acquired in a BSE mode to yield the element resolution. It is obvious that the corrosion layer was inhomogeneous. It was formed by dark and light microstructure constituents. The lighter areas point to the presence of heavier elements. The EDS point analysis is shown in Figure 11b. The light point (point 1) has a high tin and a low magnesium concentration. The darker areas (points 2 and 3) have a higher concentration of Mg. They correspond to either MgO·SnO_2_/MgSn(OH)_6_ (point 2) or MgO/Mg(OH)_2_ (point 3). The EDS results indicate that during corrosion both Mg and Sn have been oxidized.

**Figure 12:**
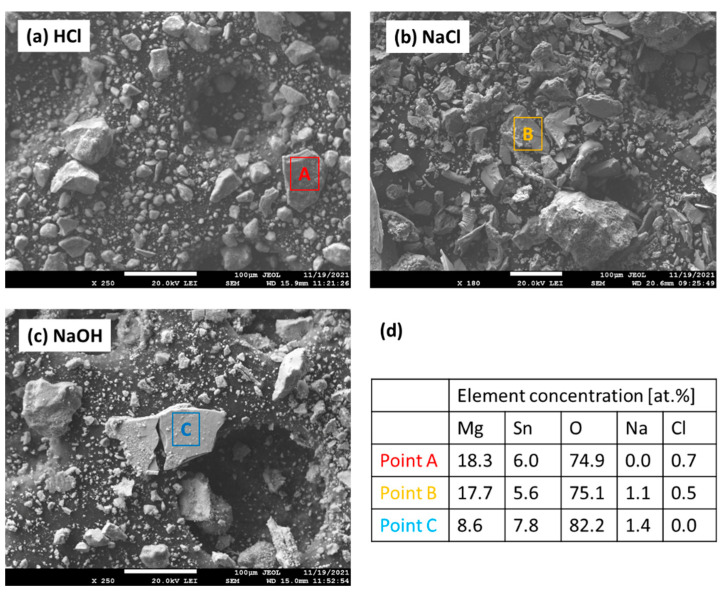
SEM images of Mg_2_Sn alloy corrosion products in NaCl (3.5 wt.%), (**a**), HCl (0.1 wt.%), (**b**) and NaOH (0.1 wt.%), (**c**). EDS analysis of points A–C is presented in (**d**).

To further investigate the chemical composition and phase constitution of the corrosion products, the Mg_2_Sn alloy was powdered. The powders were immersed in the respective electrolyte for 72 h. After reaction, the corrosion products were ground in mortar and further inspected by SEM/EDX and XRD. The microstructures of the powdered corrosion products are shown in Figure 12a–c. The EDX analyses of the individual particles are collected in Figure 12d. The EDX results show that both Mg and Sn could be found in oxidation products. In HCl and NaCl solutions, a magnesium oxide/hydroxide has been preferentially formed. Only a small amount of Sn was found. In NaOH the concentration of Sn in the corrosion product was higher compared to HCl and NaCl, respectively. Therefore, it is possible that a mixed Mg, Sn oxide/hydroxide has been formed in this electrolyte. The formation of mixed oxide/hydroxide has probably contributed to the increased protection activity of the corrosion layer in this electrolyte.

**Figure 13:**
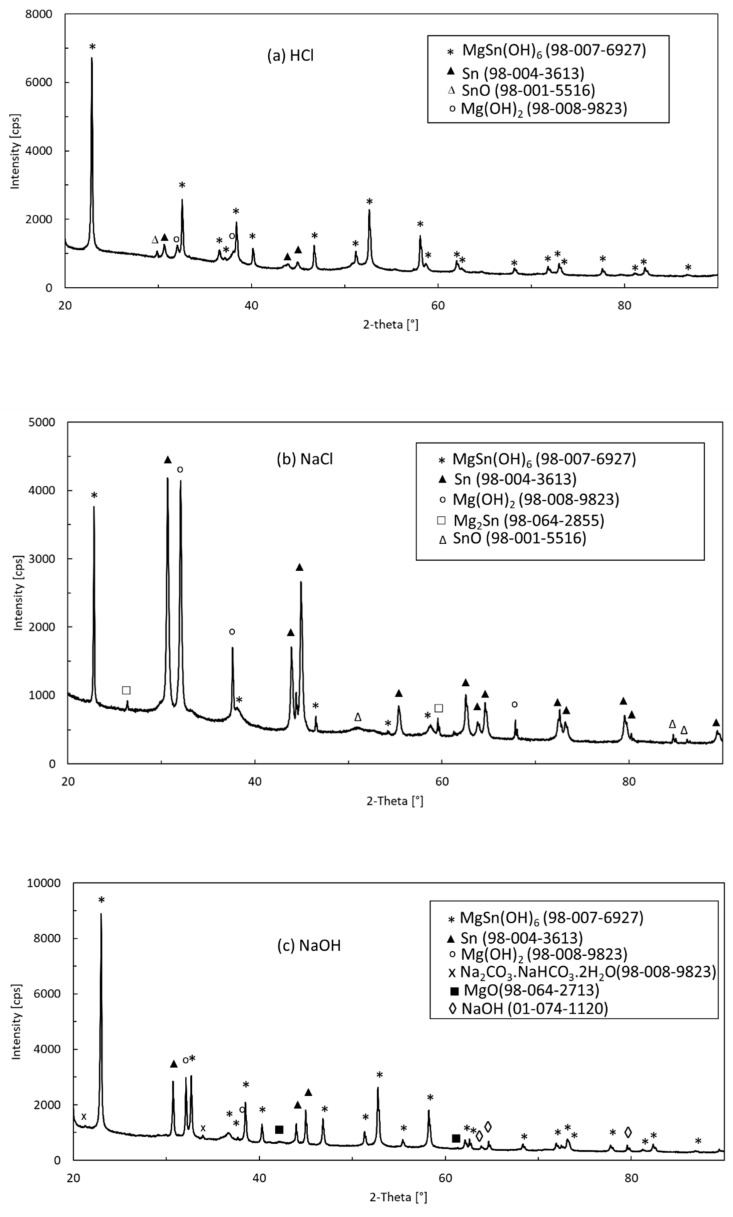
XRD patterns of the corrosion products of the Mg_2_Sn alloy formed in HCl (**a**), NaCl (**b**) and NaOH (**c**).

The EDX analysis cannot provide reliable information about the presence of light elements such as hydrogen. Therefore, the XRD measurements have been conducted to verify the phase constitution of the corrosion products. A set of XRD patterns measured for various electrolytes is presented in Figure 13. All major peaks have been assigned to the phases indicated in Table 5. To confirm the presence of the identified phases, an additional quantitative analysis was performed using the Rietveld MAUD 2.84 refinement software [58]. Phases considered in the Rietveld refinement were specified based on the ICSD FIZ Karlsruhe database. Additionally, the description of schoenfliesite (MgSn(OH)_6_) was specified based on the work of Basciano et al. [59]. In addition to the corrosion products listed in Table 5, metallic Sn was identified in all patterns (Figure 13). This observation is understandable since Sn is nobler than Mg. Mg, on the other hand, was always bound in various corrosion products. Brucite (Mg(OH)_2_) was the main corrosion product in NaCl and HCl solutions. A small amount of romarchite (SnO) was also identified in the HCl and NaCl solutions. Schoenfliesite (MgSn(OH)_6_) was the main corrosion product in NaOH and HCl. The presence of schoenfliesite caused a significant reduction in the volume fraction of metallic tin. In the alkaline electrolyte trona, sodium hydroxide and periclase were confirmed additionally.

The results indicate that schoenfliesite contributes to the increased corrosion resistance of the Mg_2_Sn alloy in alkaline electrolytes. MgSn(OH)_6_ may be formed by the following reactions:

Mg + Sn + 1.5O_2_ + 3H_2_O → MgSn(OH)_6_ (12)

SnO_2_ + 2H_2_O + Mg(OH)_2_ → MgSn(OH)_6_ (13)

In Reaction (12), schoenfliesite is formed by direct oxidation of Mg and Sn and subsequent hydration. In Reaction (13), the mixed hydroxide is formed by hydration of SnO_2_ and reaction with Mg(OH)_2_. Schoenfliesite is a cubic double hydroxide perovskite phase [60,61,62]. It is often found as a by-product during the synthesis of layered double hydroxides [63,64]. The structure of layered double hydroxides consists of positively charged [Mg(OH)_2_] octahedral layers in which part of M(II) cations are substituted by trivalent or four-valent cations. 

Previous studies indicate that Sn alloying may significantly contribute to the corrosion resistance of Mg alloys [17,18]. Sn passivates in aqueous NaCl. The passivation of Sn has already been observed on the polarization curve of this metal in Figure 8b. The corrosion products of tin may thus provide significant corrosion protection to the Mg–Sn alloys. Schoenfliesite has been reported to provide a superior oxidation resistance in Cl-containing electrolytes [65]. The stability of the Sn-rich oxide/hydroxide on magnesium is probably related to the stability of Sn(IV) species in aqueous media [45]. In chloride-containing electrolytes, magnesium hydroxide can react with Cl^−^ ions to produce OH^−^ (Reaction (5)). The OH^−^ anions can locally increase the pH in acidic and neutral electrolytes, making the formation of Sn(IV) species feasible. 

The produced Sn-rich oxide/hydroxide corrosion layer keeps the corrosion of magnesium alloys at an acceptable level. Furthermore, it provides sufficient passivation to reduce sensitivity to pitting [65,66]. The addition of stannate to the electrolyte has already been experimentally explored as an efficient way of producing protective schoenfliesite coatings on magnesium alloys by plasma electrolytic oxidation [65]. Tin in the oxide acts as an inhibitor of the dissolution reaction of magnesium. The main characteristics of the Sn-rich oxide coating are both keeping the corrosion rate of magnesium alloys at an acceptable value and providing a sufficient passivation plateau to reduce the pitting sensibility. The latter is crucial for Mg alloys as they often undergo severe galvanic corrosion in service.

## 4. Conclusions

In the present work, the microstructure, phase constitution and corrosion behavior of the Mg_2_Sn alloy have been investigated. The alloy was prepared from high purity Sn and Mg lumps by melting them in an argon atmosphere. The corrosion behavior was studied in aqueous solutions of HCl (0.1 wt.%), NaCl (3.5 wt.%) and NaOH (0.1 wt.%). The corrosion resistance was studied by hydrogen evolution, immersion and potentiodynamic experiments. The alloy was composed of intermetallic Mg_2_Sn and a small amount of Mg_2_Sn + (Sn) eutectic. The prepared alloy was nearly single phase. The volume fraction of Mg_2_Sn was 95%.

The alloy was found to be cathodic with respect to metallic Mg and anodic with respect to Sn. The corrosion potentials of the Mg_2_Sn alloy measured in HCl, NaCl and NaOH aqueous solutions were −1380, −1498 and −1361 mV vs. sat. Ag/AgCl, respectively. The highest corrosion rate of the alloy, 92 mmpy, was found in aqueous HCl. The high corrosion rate was accompanied with massive hydrogen evolution on the alloy surface. The corrosion rate was found to decrease sharply with the increasing pH of the electrolyte.

The main corrosion products formed on the alloy surface were MgSn(OH)_6_ and Mg(OH)_2_. The Sn-rich hydroxide produced in alkaline NaOH was able to provide a sufficient passivation barrier and keep the corrosion rate of the alloy at an acceptable level.

Comparison of the corrosion parameters of the present alloy with those of Mg–Sn alloys previously studied indicates that the corrosion potentials increase with increasing tin concentration. This observation shows that Sn contributes to an ennoblement of the Mg–Sn alloys. The corrosion current of the present alloy is higher compared to those of previously studied Mg–Sn alloys with significantly lower Sn atomic fractions. The Sn addition contributes to anodic activation, previously reported for Mg–Sn alloys with 1–3 at.% Sn.

Although the direct comparison of the Mg_2_Sn alloy with previously studied alloys is useful, it has certain limitations. To draw general conclusions, alloys with similar microstructure and Mg_2_Sn distribution would need to be compared. Such comparison for all Mg–Sn alloy compositions is very difficult. Furthermore, it must be noted that alloys with a Sn concentration greater than 5 at.% have been significantly less explored. As such, their corrosion investigations are underrepresented in the scholarly literature. In our future work, we aim to study the corrosion resistance of Mg–Sn alloys with larger Sn concentrations. The as-cast alloys will be further annealed to obtain equilibrium microstructures. Finer microstructures are anticipated to result in a better corrosion resistance. The good corrosion resistance may open new potential applications for Sn-rich Mg–Sn alloys in electrochemistry or soldering.

## Figures and Tables

**Table 1:** XRD measurement settings.

Sample	Angle Range (2Theta)	Incident Beam	Diffracted Beam	Detector
Powdered Mg_2_Sn alloy	20–90°	Divergence slit: 1/2° Soller slit: 0.04 rad Anti-scatter slit: 1°	Anti-scatter slit: 1° Soller slit: 0.04 rad	PIXcel3D detector in 1D scanning mode
Powdered corrosion products	20–90°	Divergence slit: 1/4° Soller slit: 0.04 rad Anti-scatter slit: 1/2°	Anti-scatter slit: 1/2° Soller slit: 0.04 rad	PIXcel3D detector in 1D scanning mode

**Table 2:** Chemical composition of points 1–5 in Figure 1 measured by EDX.

Sample	Point 1	Point 2	Point 3	Point 4	Point 5
Mg [at.%]	66.22	1.25	1.92	49.62	66.64
Sn [at.%]	33.78	98.75	98.08	50.38	33.36

**Table 3:** Phases identified during the XRD analysis of the powdered Mg_2_Sn alloy.

Chemical Formula	Reference Code–ICSD Database FIZ Karlsruhe	Crystal System	Space Group	Space Group Number	Fraction [Vol.%]
Mg_2_Sn	98-064-2855	Cubic	$Fm\bar{3}$	225	95.75
Sn	98-004-3613	Tetragonal	*I* 41*/a m d*	141	0.25
Mg_2_Sn_1.1_	98-007-0069	Hexagonal	$P\bar{3}$	147	4.00

**Table 4:** Electrochemical parameters of the as-cast Mg_2_Sn alloy in 3.5 wt.% NaCl, 0.1 wt.% HCl and 0.1 wt.% NaOH aqueous solutions at room temperature. Results for Mg and Sn measured in 3.5 wt.% NaCl are also included.

	OCP_30 min_ [mV vs. Ag/AgCl]	*E*_corr_ [mV vs. Ag/AgCl]	*j*_corr_ [A m^−2^]	*E*_pitt_ [mV vs. Ag/AgCl]
	Mg_2_Sn			
NaCl	−1522	−1498	2.10	−1461
HCl	−1393	−1380	49.0	−1255
NaOH	−1311	−1361	0.15	-
	Mg			
NaCl	−1592	−1697	1.40	−1551
	Sn			
NaCl	−441	−479	0.012	−358

**Table 5:** Product phases and their relative volume fractions identified during XRD analysis of Mg_2_Sn alloy powders after corrosion. Legend: ■■■■: dominant corrosion product; ■■: lower relative amount; ■: low degree of certainty; -: not detected.

Compound Name	Formula	HCl	NaCl	NaOH
Schoenfliesite	MgSn(OH)_6_	■■■■	■■	■■■■
Brucite	Mg(OH)_2_	■■	■■■■	■
Romarchite	SnO	■	■	-
Periclase	MgO	-	-	■

## Data Availability

Data are available from the corresponding author upon request.
